# Characteristics and Predictive Value of Blood Transcriptome Signature in Males with Autism Spectrum Disorders

**DOI:** 10.1371/journal.pone.0049475

**Published:** 2012-12-05

**Authors:** Sek Won Kong, Christin D. Collins, Yuko Shimizu-Motohashi, Ingrid A. Holm, Malcolm G. Campbell, In-Hee Lee, Stephanie J. Brewster, Ellen Hanson, Heather K. Harris, Kathryn R. Lowe, Adrianna Saada, Andrea Mora, Kimberly Madison, Rachel Hundley, Jessica Egan, Jillian McCarthy, Ally Eran, Michal Galdzicki, Leonard Rappaport, Louis M. Kunkel, Isaac S. Kohane

**Affiliations:** 1 Informatics Program at the Harvard–Massachusetts Institute of Technology Division of Health Sciences and Technology, Boston Children's Hospital, Boston, Massachusetts, United States of America; 2 Division of Genetics, Program in Genomics, Boston Children's Hospital, Boston, Massachusetts, United States of America; 3 Manton Center for Orphan Disease Research, Boston Children's Hospital, Boston, Massachusetts, United States of America; 4 Division of Developmental Medicine, Boston Children's Hospital, Boston, Massachusetts, United States of America; 5 Department of Psychiatry, Boston Children's Hospital, Boston, Massachusetts, United States of America; 6 Department of Cardiology, Boston Children's Hospital, Boston, Massachusetts, United States of America; 7 Department of Pediatrics, Harvard Medical School, Boston, Massachusetts, United States of America; 8 Howard Hughes Medical Institute, Harvard Medical School, Boston, Massachusetts, United States of America; 9 Department of Genetics, Harvard Medical School, Boston, Massachusetts, United States of America; 10 Center for Biomedical Informatics, Harvard Medical School, Boston, Massachusetts, United States of America; 11 Autism Consortium, Boston, Massachusetts, United States of America; University of Jaén, Spain

## Abstract

Autism Spectrum Disorders (ASD) is a spectrum of highly heritable neurodevelopmental disorders in which known mutations contribute to disease risk in 20% of cases. Here, we report the results of the largest blood transcriptome study to date that aims to identify differences in 170 ASD cases and 115 age/sex-matched controls and to evaluate the utility of gene expression profiling as a tool to aid in the diagnosis of ASD. The differentially expressed genes were enriched for the neurotrophin signaling, long-term potentiation/depression, and notch signaling pathways. We developed a 55-gene prediction model, using a cross-validation strategy, on a sample cohort of 66 male ASD cases and 33 age-matched male controls (P1). Subsequently, 104 ASD cases and 82 controls were recruited and used as a validation set (P2). This 55-gene expression signature achieved 68% classification accuracy with the validation cohort (area under the receiver operating characteristic curve (AUC): 0.70 [95% confidence interval [CI]: 0.62–0.77]). Not surprisingly, our prediction model that was built and trained with male samples performed well for males (AUC 0.73, 95% CI 0.65–0.82), but not for female samples (AUC 0.51, 95% CI 0.36–0.67). The 55-gene signature also performed robustly when the prediction model was trained with P2 male samples to classify P1 samples (AUC 0.69, 95% CI 0.58–0.80). Our result suggests that the use of blood expression profiling for ASD detection may be feasible. Further study is required to determine the age at which such a test should be deployed, and what genetic characteristics of ASD can be identified.

## Introduction

Autism Spectrum Disorders (ASD) cover a broad spectrum of developmental delays in social interaction, verbal and non-verbal communication, and restricted repetitive patterns of behavior and interests with onset before 3 years of age. ASDs include autistic disorder, pervasive developmental disorder-not otherwise specified and Asperger's Disorder as sub classified in the *Diagnostic and Statistical Manual of Mental Disorders, 4th edition, Text Revision* (DSM-IV-TR) [1]. The prevalence of ASD has been reportedly increasing in recent decades, with a current estimation at 1 in 88 [2]. There are long waiting lists for evaluation at most centers with expertise and, despite the progress made in adopting instruments such as the Autism Diagnostic Interview-Revised (ADI-R) and the Autism Diagnostic Observation Schedule (ADOS), there remains significant debate regarding the prognostic value and accuracy of existing instruments [3], [4]. Additionally, the Centers for Disease Control have identified addressing the delay in diagnosis of ASD (median age at diagnosis is currently 5.7 years) as a public health priority [5], [6]. Moreover, early diagnosis and behavioral intervention improve outcomes [7], highlighting a continued need and interest in diagnostic tests or biomarkers that can be used in primary pediatric care to reduce the time to accurate diagnosis.

The high heritability of ASD, with 60–90% concordance between identical twins vs. 0–10% in fraternal twins [8], [9], has led to the hope that a collection of DNA mutations can be used diagnostically for ASD. Indeed, a range of mutations, from single nucleotide changes to copy number variants (hundreds to millions of bases affected) to karyotypically visible anomalies, have been catalogued in patients with ASD. However, individually most of these mutations account for less than 1% of autism cases and collectively they account for less than 20% [8]. Chromosomal microarray analysis (CMA), which detects 7–10% of children diagnosed with ASD [10]–[12], has been recommended as a first-tier genetic test for patients who may have ASD. Although DNA sequence and chromosomal variants may provide mechanistic insight, CMA characterizes genomic variants in only a minority of children with ASD.

Gene expression microarrays enable the measurement of messenger RNA for most of the thousands of known genes. Specifically, they measure which part of the DNA in the genome is transcribed for cellular function at a given time. Multivariate gene expression–based prediction models developed from cases and non-cases have been widely used for diagnosis, screening, prediction of treatment response, and prognosis [13], [14]. RNA expression, across hundreds of genes in peripheral blood, has also been shown to be perturbed in patients with ASD relative to controls using gene expression microarrays [15]–[23]. How these RNA expression differences translate into classification accuracy is not yet known. Nonetheless, as RNA expression is controlled by both the DNA code from which it is transcribed and the physiological and environmental milieu, these early results are encouraging. We performed the largest blood gene expression study to date of ASD, designed specifically to provide insight into the performance of a blood expression signature that classifies children with ASD from controls, particularly after an increased index of suspicion based on parent and/or pediatric assessment. Validation of this signature utilized an additional cohort for assessment of classification accuracy.

## Results

ASD patients were recruited from the Developmental Medicine Center, the Division of Genetics, and the Department of Neurology at Children's Hospital Boston (CHB) with additional samples obtained from Boston Medical Center, Cambridge Health Alliance, Tufts Medical Center, and Mass General Hospital in collaboration with the Autism Consortium of Boston. Study inclusion criteria consisted of a clinical diagnosis of ASD by DSM-IV-TR criteria and an age>24 months. Patients with ASD recruited for this study have undergone diagnostic assessment, using ADOS and ADI-R, as well as comprehensive clinical testing such as cognitive testing, language measures, medical history, height and weight, head circumference, and behavioral questionnaires. Two independently collected data sets (hereafter P1 and P2) consisted of 99 (66 ASDs and 33 controls) and 186 (104 ASDs and 82 controls) individuals, respectively. The patients with known syndromic disorders such as fragile X syndrome, tuberous sclerosis, Landau-Kleffner syndrome, and Klinefelter syndrome were not included in this study.

A total of 115 controls were enrolled concurrently. Collection of control samples was performed through partnerships with both the Division of Endocrinology of Boston Children's Hospital (12 individuals from the P1 cohort) and Children's Hospital Primary Care Center (CHPCC) (21 individuals from P1, and all 82 individuals from P2). Patients enrolled from the outpatient endocrine clinic were healthy children with idiopathic short stature, including genetic short stature and constitutional delay of growth, and were having clinical blood draws. We followed up on the clinical blood draw results to confirm they had no abnormal findings and those that did were withdrawn from the study. Patients seen in the CHPCC for a well-child visit that involved a routine blood draw (for example, to obtain lead levels) were offered enrollment. A diagnosis of a chronic disease, intellectual disability, ASD, or other neurological disorder acted as exclusion criteria from our control group. Complete phenotypic information is available with microarray data (Gene Expression Omnibus identifier GSE18123). Each cohort's clinical and demographic information is shown in Table 1.

**Table 1:** Characteristics of patients with autism spectrum disorders and controls in the training set (P1) and in the validation set (P2).

	Training Set (P1)		Validation Set (P2)	
Characteristic	ASD	Control	ASD	Control
No.	66	33	104	82
Age - years				
Mean	8.0	9.0	8.4	8.1
Interquartile range	5.5–9.7	4.0–13.1	5.0–11.0	4.1–12.3
Male - no. (%)	66 (100)	33 (100)	80 (77)	48 (59)
Diagnosis (Male %)				
Autistic Disorder	31	-	40 (75)	-
PDD, NOS	26	-	49 (76)	-
Asperger's Disorder	9	-	15 (87)	-
Race - no.				
Caucasian	60	13	96	33
Black	0	5	0	8
Asian	1	1	3	2
Mixed	5	1	4	8
Other	-	4	-	21
Unknown	1	9	1	10
Ethnicity				
Hispanic - no.	2	9	8	36
Unknown - no.	1	-	-	-
Developmental delay - no.	21	5	51	3
Learning Disorder – no.	9	-		-
Psychiatric Disorder - no.	14	4	32	1
Neurological Disorder - no.	8	-	18	-
Gastrointestinal Disorder - no.	24	-	20	-
Autoimmune Disorder - no.	-	-	7	-
Cerebral Palsy - no.	-	-	1	-

Written consent was obtained from the parent or guardian of all children participating in the study, and was approved by the Institutional Review Boards (IRB) of each participating institution. Approval for the study as a whole was also obtained from the Boston Children's Hospital IRB.

There was no statistical difference in age between ASD cases and controls in the P1 (Welch's t-test *P* = 0.29) or P2 cohort (*P* = 0.73). Ages of ASD samples between the P1 and P2 populations were also not different (*P* = 0.52). Thirteen of 66 patients with ASD in P1 and 42 out of 104 in P2 were evaluated for verbal and non-verbal IQ. There was no significant difference in average IQs between P1 and P2 (verbal IQ *P* = 0.872, non-verbal IQ *P* = 0.624, and total IQ *P* = 0.929). One ASD patient in P1 met the criteria of mild intellectual disability (verbal IQ = 69, non-verbal IQ = 65, and total IQ = 67), and 5 males and 2 females of P2 met the criteria of moderate to profound intellectual disability.

The disease incidence in ASD is discordant between males and females, with males 4 times more likely to develop disease. Additionally, our preliminary analysis revealed higher heterogeneity in RNA levels in females with ASD than in males, possibly due to the smaller number of females included in this study or to the sexual dimorphism in the expression of the disorder [17]. Considering these factors, only males were included in the P1 cohort (both ASD and control samples), which was used to build a prediction model for ASD. We subsequently tested the performance of the predictive model in both males and females in the P2 cohort (although the number of female controls was higher than that of female ASD—Fisher's exact test *P* = 0.01 in P2).

### Blood gene expression changes in ASD

Due to the time-span covered by this study, expression studies were performed by microarray profiling using an earlier version of the Affymetrix array (U133p2) for the P1 data set and a later version (GeneST) for the P2 data set. After selecting the best matching probesets between the two platforms (see Methods), principal component analysis was performed to project samples into the first two principal components. P1 and P2 samples did not form two clusters after combining the two datasets, which were centered and scaled independently (Fig. S1) [13].

There were 489 and 610 transcripts differentially expressed between ASD cases and controls in the P1 and P2 datasets, respectively (Welch's t-test *P*<0.001, corresponding FDRs 0.029 (P1), and 0.023 (P2)) (Tables S1 and S2). Of these, 23 genes—*ARID4B, ARMCX3, C10orf28, CTBP2, DDX3Y, JRKL, MTERFD3, NFYA, NGEF, PNN, RLF, RNF145, TIGD1, TUBB2A, UTY, YES1, ZNF117, ZNF322, ZNF445, ZNF514, ZNF518B, ZNF540*, and *ZNF763*—were significant in both cohorts. To calculate the significance of this overlap, we shuffled sample labels in both data sets 200,000 times and counted the number of permutations with as many or more overlapping genes. Out of 200,000 permutations, only 2 had at least 23 overlapping genes between the two data sets, yielding a permutation *P* = 10^−5^. The overlap of 23 genes also showed a significant trend using the hypergeometric distribution (P = 0.0721) [24]. In the P2 dataset, 352 genes were significant for male patients compared to male controls while 48 genes were significant for female groups (Welch's t-test P<0.001, corresponding FDRs 0.028 (P2 males) and 0.60 (P2 females)). One gene – *POLR3H* – was differentially expressed in both males and females.

Twelve of the 489 differentially expressed genes in the P1 dataset were selected for validation by quantitative RT-PCR. The 12 genes selected had an average fold change between ASD and controls greater than 1.5 and a mean expression level on the array greater than 150. These were *CREBZF, HNRNPA2B1, KIDINS220, LBR, MED23, RBBP6, SPATA13, SULF2, TMEM30A, ZDHHC17, ZMAT1*, and *ZNF12*. Eleven of 12 genes (all except *ZMAT1*) were successfully validated using qRT-PCR (Table 2).

**Table 2:** Quantitative RT-PCR validations of 12 differentially expressed genes.

		qRT-PCR		Microarray	
Gene	TaqMan Primer ID	Fold change	p-value	Fold change	p-value
*CREBZF*	Hs02742201_s1	1.73	0.000127974	1.60	8.8516E-05
*HNRNPA2B1*	Hs00955384_m1	1.35	0.00119253	1.53	4.2587E-06
*KIDINS220*	Hs01057000_m1	2.16	8.44446E-10	1.57	2.674E-05
*LBR*	Hs01032700_m1	2.50	7.55278E-10	1.63	5.85338E-05
*MED23*	Hs00606608_m1	2.24	1.95917E-09	1.51	0.000259037
*RBBP6*	Hs00544663_m1	1.98	0.000388767	1.58	0.000156489
*SPATA13*	Hs01128069_m1	1.61	0.000236786	1.56	6.07308E-05
*SULF2*	Hs01016476_m1	1.89	5.58742E-08	1.72	7.35118E-06
*TMEM30A*	Hs01092148_m1	3.19	4.27915E-10	1.84	7.26489E-05
*ZDHHC17*	Hs00604479_m1	3.82	7.3983E-12	1.61	1.22144E-05
*ZMAT1*	Hs00736844_m1	0.60	0.413889282	1.86	8.81564E-05
*ZNF12*	Hs00212385_m1	2.35	9.12987E-09	1.54	1.86789E-06

We selected 12 significantly differentially expressed genes that had average fold change greater than 1.5 and mean expression levels greater than 150 in the P1 dataset, and validated changes using quantitative RT-PCR. A total of 30 ASD and 30 control samples from the P1 population were run in replicates of four on the Biomark real time PCR system (Fluidigm, CA) using nanoliter reactions and the Taqman system (Applied Biosystems, CA). We were limited to 60 samples because the other 39 samples did not have enough RNA for qRT-PCR. The housekeeping gene used for qRT-PCR normalization was *GAPDH* (Hs9999905_m1). The values shown are for 30 ASD and 30 control samples from the P1 population, and fold changes refer to ASD/Control. P-values were calculated using Welch's t-test. For microarray data, p-values and fold changes were recalculated using the available samples. Eleven of 12 genes (all except *ZMAT1*) were successfully validated.

Out of 489 differentially expressed genes in P1, 10 genes (*AFF2, CD44, CNTNAP3, CREBBP, DAPK1, JMJD1C, NIPBL, PTPRC, SH3KBP1*, and *STK39*) were found in the expert-curated ASD candidate-genes database (https://gene.sfari.org/) [25]. Additionally, 44 genes mapped to reported copy number variation regions (http://projects.tcag.ca/autism/) (Table 3) [12]. Interestingly, rare mutations in or CNVs containing *JMJD1C* [26], *PTPRC* [27], and *SH3KBP1* [28] have been reported in a small numbers of cases. For example, *STK39* was identified as an ASD candidate gene from linkage analysis of 334 families [29]. Two genes—*CD44* and *DAPK1*—were differentially expressed between 5 monozygotic twins pairs who were discordant for clinical severity [30]. *AFF2, DOCK8*, *NIPBL*, and *RPS6KA3* were implicated in intellectual disability. *AFF2* encodes FRAXE-associated mental retardation protein (FMR2) within which small changes were found in patients with intellectual disability and developmental delay [31], and significantly more frequent rare variants were detected in *AFF2* by massively parallel sequencing of males with ASD [32]. Heterozygous changes in the *DOCK8* gene have been previously reported in two unrelated patients, one by deletion testing and one by a translocation breakpoint; these disruptions are associated with intellectual disability and developmental disability (*MRD2*, MIM ID# 614113) [33]. Mutations in *NIPBL* result in Cornelia de Lange syndrome (MIM ID# 122470), a disorder characterized by dysmorphic facial features, growth delay, limb reduction defects as well as intellectual disability [34]. Among the differentially expressed genes in the P2 dataset, only *ATRX* was associated with intellectual disability according to the Online Mendelian Inheritance in Man (OMIM) database [35].

**Table 3:** Differentially expressed genes in CNV regions previously linked to ASD.

Copy number variation	Differentially expressed genes in P1 dataset
Gain	*ADAM10, AP1G1, CCNL1, CLIP1, DDX55, DOCK8, GRIPAP1, HIPK3, JMJD1C, KLHL2, MAPK8, MTMR10, PCGF3, RNF111, SACS, SNX27, SPATA13, TAOK3, WDR7, ZNF268, ZZEF1*
Loss	*ANTXR2, ATRN, FRMD4B, HECA, ING5, LIFR, OR10A4, SIN3A, UTRN, VAV3, ZC3H13, ZNF548, ZNF592*
Gain and loss	*AHR, CRKL, DMXL1, KBTBD11, KIAA0947, KIAA1468, MAPK1, TRIO, ZBED4, ZNF516*

When each diagnostic subtype was compared to controls in the P1 dataset, 178, 56, and 3 genes were significant for autistic disorder (AUT), pervasive developmental disorder-not otherwise specified (PDDNOS), and Asperger's disorder (ASP), respectively (One-way analysis of variance (ANOVA) with Dunnett's *post hoc* test *P*<0.001, corresponding FDRs 0.076 (AUT), 0.24 (PDDNOS), and 1.0 (ASP)). Among the significant genes in ASP, only one gene, *PTPRE*, overlapped with the AUT vs. control or PDDNOS vs. control comparisons while 36 genes were in common between AUT vs. control and PDDNOS vs. control (Fig. S2).

Four of 66 ASD cases in the P1 dataset had mild intellectual disability. When we compared the 4 ASD cases with mild intellectual disability to the 62 ASD cases without intellectual disability, we found 70 differentially expressed genes (P<0.001, corresponding FDR 0.12), of which none has yet been implicated in the intellectual disability process as reported in the OMIM and Human Gene Mutation Databases [36]. The relation between ASD and intellectual disability needs to be further explored in the context of the genetic background that they share.

Expression profiling also identified chromosomal abnormalities. For instance, we identified an affected male that had high expression of the X-inactive-specific transcript (*XIST*); the expression values were comparable to those of females. Subsequent karyotyping confirmed Klinefelter syndrome in this individual, and the case was excluded in this study for further analysis.

### Perturbed biological pathways and identification of heterogeneous subgroups

We used a modified Fisher's exact test (i.e., Expression Analysis Systematic Explorer [EASE] score) to see what biological pathways were enriched with the differentially expressed genes in P1 using the DAVID functional annotation system [37], [38]. This metric allowed us to calculate which processes were overrepresented in the 489 differentially expressed genes in P1 relative to all the processes annotated in the Kyoto Encyclopedia of Genes and Genomes (KEGG) [39]. These results are detailed in Table 4. In brief, the neurotrophin signaling pathway (KEGG pathway identifier: hsa04722) was the most significant (EASE score P = 0.00023, FDR 0.0026) among 22 overrepresented pathways (EASE score *P*<0.05, corresponding FDR 0.44). The neurotrophin signaling pathway includes neurotrophins and their second messenger systems such as the MAPK pathway, PI3K pathway, and PLC pathway, which have been identified by others [40], [41] as important for neural development, learning and memory, and syndromic ASDs such as tuberous sclerosis and Smith-Lemli-Opitz syndrome. Interestingly, long-term potentiation and long-term depression pathways were also significant (EASE score *P* = 0.011, FDR 0.11, and *P* = 0.042, FDR 0.39 respectively). We grouped the 22 overrepresented pathways according to the number of shared genes by calculating Cohen's kappa score. Two enriched clusters of 15 and 3 pathways were significant (Cohen's kappa>0.5) with progesterone-mediated oocyte maturation belonging to both clusters. Five other pathways—notch signaling pathway, lysosome, leukocyte transendothelial migration, endocytosis, and MAPK signaling pathway—were not clustered with the others (Table 4).

**Table 4:** Top 22 KEGG pathways enriched for differentially expressed genes in ASD (P1).

KEGG pathways	Count	EASE score P	FDR (%)	Genes
**Pathway Cluster 1**				
Neurotrophin signaling pathway	13	0.00023	0.26	*MAP2K1, PIK3CB, PIK3CD, KIDINS220, MAPK1, YWHAG, MAP3K5, RPS6KA3, CRKL, MAPK14, SH2B3, MAPK8, CRK*
Fc gamma R-mediated phagocytosis	9	0.00303	3.41	*MAPK1, PTPRC, DOCK2, CRKL, VAV3, MAP2K1, PIK3CB, PIK3CD, CRK*
Renal cell carcinoma	8	0.00307	3.45	*MAPK1, CRKL, MAP2K1, PIK3CB, PIK3CD, CREBBP, EGLN1, CRK*
Chemokine signaling pathway	12	0.01094	11.82	*MAPK1, DOCK2, CRKL, VAV3, ROCK1, MAP2K1, GNAI1, PIK3CB, PREX1, PIK3CD, CCR2, CRK*
Regulation of actin cytoskeleton	14	0.01174	12.62	*GNA13, VAV3, MAP2K1, ROCK1, PIK3CB, PIK3CD, SSH2, IQGAP2, ITGB2, MAPK1, CRKL, ITGAV, PPP1R12A, CRK*
mTOR signaling pathway	6	0.01358	14.47	*MAPK1, RPS6KA3, PIK3CB, PIK3CD, CAB39, RICTOR*
Chronic myeloid leukemia	7	0.01413	15.01	*MAPK1, CRKL, CTBP2, MAP2K1, PIK3CB, PIK3CD, CRK*
Fc epsilon RI signaling pathway	7	0.02189	22.35	*MAPK1, VAV3, MAP2K1, PIK3CB, MAPK14, PIK3CD, MAPK8*
B cell receptor signaling pathway	6	0.02773	27.48	*MAPK1, VAV3, MAP2K1, PIK3CB, PIK3CD, PPP3CB*
T cell receptor signaling pathway	8	0.02797	27.69	*MAPK1, PTPRC, VAV3, MAP2K1, PIK3CB, MAPK14, PIK3CD, PPP3CB*
Focal adhesion	12	0.02878	28.38	*IGF1R, MAPK1, CRKL, VAV3, ROCK1, MAP2K1, PIK3CB, ITGAV, PIK3CD, PPP1R12A, MAPK8, CRK*
ErbB signaling pathway	7	0.02987	29.29	*MAPK1, CRKL, MAP2K1, PIK3CB, PIK3CD, MAPK8, CRK*
Natural killer cell mediated cytotoxicity	8	0.04051	37.66	*IFNAR2, MAPK1, VAV3, MAP2K1, PIK3CB, PIK3CD, PPP3CB, ITGB2*
VEGF signaling pathway	6	0.04888	43.6	*MAPK1, MAP2K1, PIK3CB, MAPK14, PIK3CD, PPP3CB*
**Pathway Cluster 1 and 2**				
Progesterone-mediated oocyte maturation	9	0.00408	4.57	*IGF1R, MAPK1, RPS6KA3, MAP2K1, GNAI1, PIK3CB, MAPK14, PIK3CD, MAPK8*
**Pathway Cluster 2**				
Long-term potentiation	7	0.01054	11.4	*MAPK1, RPS6KA3, GNAQ, MAP2K1, CREBBP, PPP3CB, PPP1R12A*
Long-term depression	6	0.04209	38.82	*GNA13, IGF1R, MAPK1, GNAQ, MAP2K1, GNAI1*
**Not clustered**				
Notch signaling pathway	6	0.00536	5.96	*CTBP2, KAT2B, MAML1, CREBBP, ADAM17, MAML3*
Lysosome	9	0.01136	12.24	*LAMP1, NPC1, AP1G1, HEXB, GAA, CTSD, PPT1, CLTC, MANBA*
Leukocyte transendothelial migration	9	0.0174	18.18	*RASSF5, VAV3, ROCK1, GNAI1, PIK3CB, MAPK14, PIK3CD, PECAM1, ITGB2*
Endocytosis	11	0.02135	21.85	*EPS15, IGF1R, RNF103, RAB22A, RAB5A, GIT2, SH3KBP1, PDCD6IP, CLTC, ARAP2, ARAP1*
MAPK signaling pathway	14	0.04635	41.86	*MAP2K1, NLK, TAOK3, PPM1B, MAP4K4, MAPK1, MAP3K5, RPS6KA3, CRKL, MAPK14, PPP3CB, MAPK8, CRK, RASA1*

**Figure 1:**
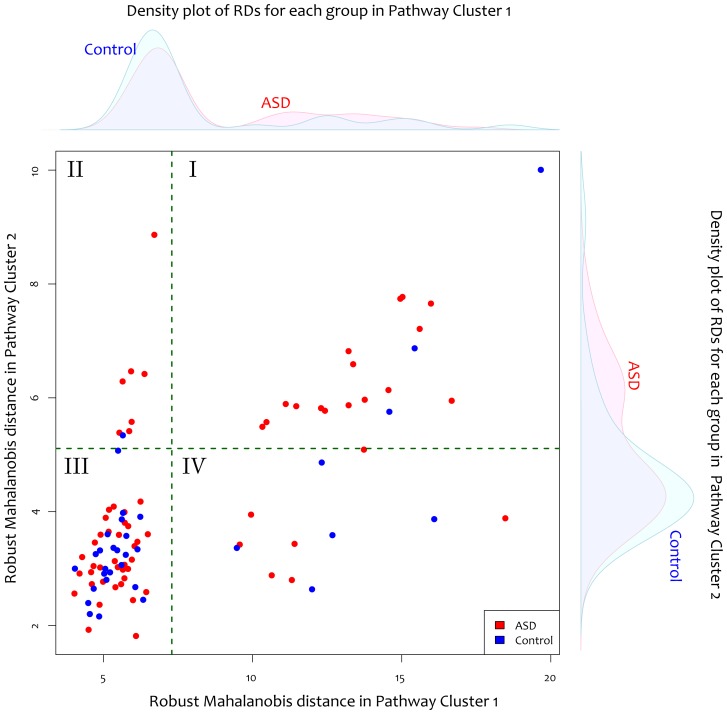
Heterogeneous subgroups in dysregulated pathways. For immune response and synaptic gene sets, robust Mahalanobis distances (RDs) were calculated for all P1 samples. The outlier cutoff was set at the 97.5% quantile of the chi-squared distribution for each gene set (dotted green lines). When all samples were plotted in the 2-dimensional plane of Pathway Cluster 1 (x axis) by RDs in the Pathway Cluster 2 (y axis) (Table 4), four subgroups of samples were distinct. Both gene sets were perturbed for the samples in quadrant I; however, the samples in quadrants II and IV were significant for one gene set but not the other. A majority of samples were in quadrant III where no significant perturbation was found. The marginal density plots show the RD distributions for each gene set. Twenty-three out of 66 ASD samples (34.8%) were outliers for the synaptic gene set compared to 4 of 33 for controls (12.1%) (Fisher's exact test *P* = 0.017). For the immune response gene set, outliers were not biased towards case or control (Fisher's exact test *P* = 0.36).

Given that multiple pathways were significantly enriched with the differentially expressed genes, we investigated the heterogeneity of perturbation across samples. All the significant genes in the top 14 pathways, from neurotrophin signaling to the VEGF pathway (Table 4), were grouped together as Pathway Cluster 1. A majority of these genes were associated with immune response. The genes in the long-term potentiation and long-term depression pathways were grouped as Pathway Cluster 2. In this cluster, synaptic genes were enriched. When the samples were plotted in a multidimensional space corresponding to the two pathway clusters (Fig. 1), four subgroups were distinct. The samples in quadrant I of Figure 1 were perturbed in both Pathway Cluster 1 and Pathway Cluster 2, while the majority of samples in quadrant III were not significantly perturbed for either gene set. Interestingly, a subgroup of ASD samples was only perturbed for Pathway Cluster 2 (quadrant II in Fig. 1), and some were only significant for Pathway Cluster 1 (quadrant IV in Fig. 1). We also found 6 significant clusters of Gene Ontology biological process terms grouped by the same approach as KEGG pathways (Cohen's kappa>0.5) from 428 overrepresented terms (Table S3), but the heterogeneity in these terms was not as clear as in KEGG pathways.

### Prediction of autism using blood gene expression signatures

To test whether peripheral blood gene expression profiles could be used as a molecular diagnostic tool for identifying ASD, we used a repeated leave-group out cross-validation (LGOCV) strategy with P1 to build a prediction model. First, the training set (P1) was utilized to determine a classification signature (i.e. a combination of gene expression measurements) that was used to classify ASD patients in P1 (compared to controls). We ranked the 489 differentially expressed genes according to their area under the receiver operating characteristic (ROC) curve (AUC). Next we excluded those genes with low expression, requiring the minimum expression level across all samples to be at least 150. A total of 391 differentially expressed genes were then utilized in building the prediction models, which were subsequently tested against the samples in our independent validation cohort (P2). The top N genes (where N ranges from 10 to 390 incremented by 5) were used to build prediction models using a repeated 5-folds LGOCV with a partial least squares (PLS) method [42], [43], and AUCs were calculated for each cross-validation instance (see Methods). The prediction model using the top 55 genes was the most stable from 100-repeated LGOCV, having the smallest coefficient of variation in AUCs from 100 trials (Fig. S3). The top 55 genes performed significantly better than the 50-gene model (one sided t test *P* = 0.00031). We chose the 55-gene prediction model because it minimized description length—i.e., the number of predictor genes—while maintaining good prediction performance, and used it to evaluate the independent dataset, P2. The 55 significant genes are listed in Table S4. The performance of PLS was comparable to that of other prediction algorithms (Table S5); thus the classification performance was not attributable to a specific prediction algorithm.

**Figure 2:**
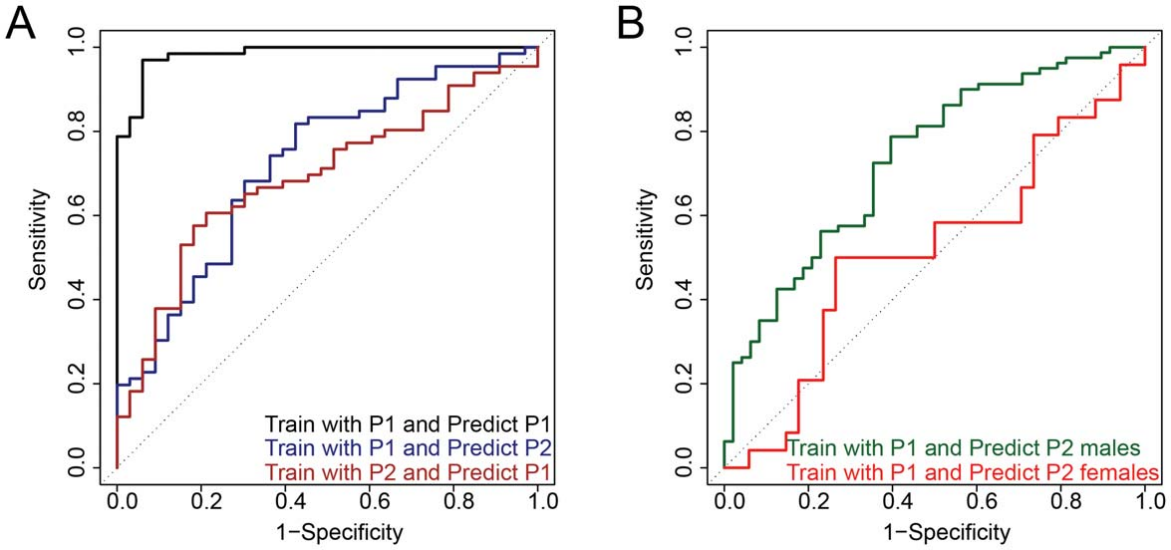
Performance of the ASD55 prediction model. Receiver operating characteristic (ROC) curve analysis was performed to evaluate the prediction accuracy. The dotted diagonal line represents random classification accuracy (AUC 0.5). **A**. The accuracy of ASD55 within P1 was unsurprisingly high (AUC 0.98, 95% confidence interval (CI), 0.965–1.000, black ROC curve). The ASD55 model was trained with P1 to predict the diagnosis of each sample in an independently collected dataset P2 (dark blue ROC curve). The performance measured by AUC was 0.70 (95% CI, 0.62–0.77). ASD55 genes showed similar performance when the training and testing datasets were switched (AUC 0.69, 95% CI 0. 58–0.80, brown ROC curve). **B**. P2 male samples were accurately predicted (dark green) while female samples (red) were not (AUC 0.73 and 0.51 respectively) when the ASD55 model was trained with P1.

The accuracy of this 55-gene set (hereafter referred to as ASD55) within P1 was unsurprisingly high since it was the training set (AUC 0.98, 95% confidence interval (CI), 0.965–1.000), but ASD55 also had good performance when applied to the P2 validation population (AUC 0.70, 95% CI 0.623–0.773) (Table 5). When generating a set of genes to classify samples, a tradeoff between specificity and sensitivity must be considered to achieve optimal results as shown by the ROC curves in Fig. 2A. To address whether the ASD55 classifier performed better than expected by chance, 55 genes were randomly sampled 2,000 times and the performances of these random sets were evaluated by AUCs. Our ASD55 model outperformed all of the 2,000 trials of randomly chosen sets of 55 genes (permutation *P*<0.0005). Since the majority of our training set (P1) consisted of ASD patients, we checked if the performance of ASD55 was inflated from such imbalances by calculating the ‘balanced accuracy’ [44]. The balanced accuracy is defined as the average of the accuracies obtained in either class (patients and control), or, equivalently, the arithmetic mean of specificity and sensitivity. It is equal to the conventional accuracy if the classifier performs equally well on both classes, but if the classifier's accuracy is entirely due to imbalance in the data the balanced accuracy will drop to random chance (0.5). The average balanced accuracy of ASD55 within P1 was 0.72, which is higher than random chance (0.5) implying that ASD55 was not entirely affected by imbalanced data [44]. Our training set (P1) consisted of males only while the test set (P2) had both genders. Unsurprisingly, the prediction model built with males performed better for males in P2. The AUC for male samples in P2 was 0.73 (95% CI 0.645–0.824) compared to 0.51 (95% CI 0.357–0.672) for female samples. To test the robustness of ASD55, we trained ASD55 with P2 samples to classify P1 samples, switching our training and validation sets. The performance was comparable to the original classification accuracy where P1 was used as the training set (AUC 0.69, 95% CI 0.583–0.797, Fig. 2B). All male patients with intellectual disability were accurately classified in both training and validation datasets while two female cases were predicted as non-cases.

**Table 5:** Prediction performance of ASD55 trained with P1.

Validation Set	AUC (95% Confidence Intervals)	Accuracy (%)	Sensitivity (%)	Specificity (%)	Positive Predictive Value (%)	Negative Predictive Value (%)
P2	0.70 (0.623–0.773)	67.7	69.2	65.9	72.0	62.8
P2 (male)	0.73 (0.645–0.824)	72.7	90.0	43.8	72.7	72.4
P2 (female)	0.51 (0.357–0.672)	63.8	50.0	73.5	57.1	67.6

Abbreviations: ASD55, the genes in a classifier developed on P1 with 55 genes listed in Table S4; AUC, area under the receiver operating characteristic curve.

**Figure 3:**
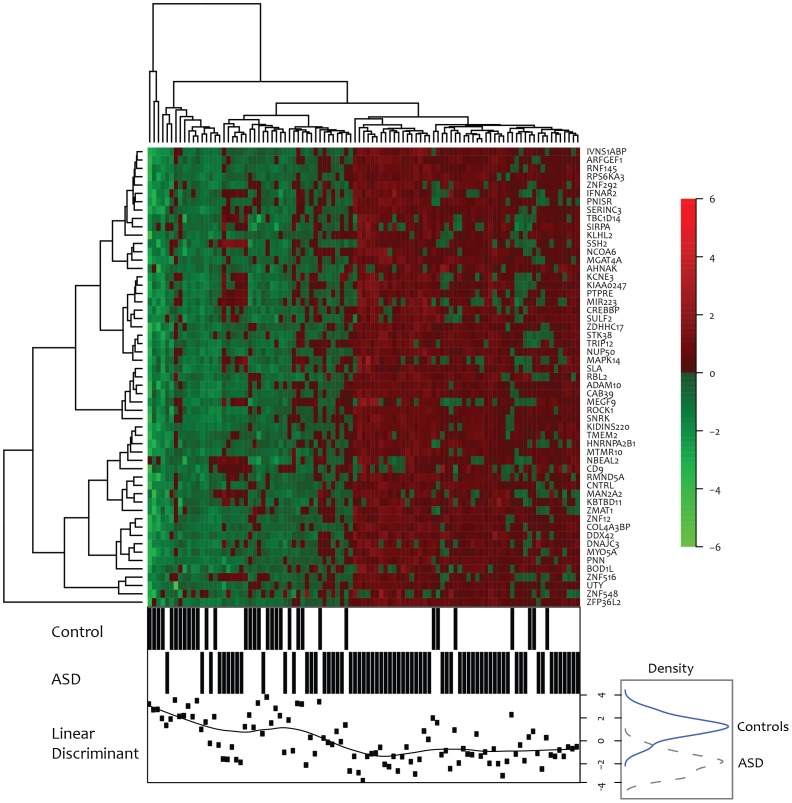
Cluster analysis of the 55 genes used in the prediction model (ASD55). The dendrogram and heatmap on top show hierarchical clustering (average linkage) of the 99 samples in the training set (P1) and the 55 genes used in our prediction model. The first 2 lines in the graph on bottom indicate whether each sample is from the patient group or the control group. Finally, the bottom line shows the distribution of Fisher's linear discriminant scores (dots) based on ASD55 with moving average (line). The distributions of linear discriminant scores are shown on the right (blue solid line for controls and black broken line for patients). ASD cases and controls are well separated using linear discriminant analysis on the ASD55 genes.

Overall, the ASD55 predictor genes were enriched with 2 KEGG pathways (TGF-beta signaling pathway and Neurotrophin signaling pathway) and 8 Gene Ontology biological process terms (Table S6). It may be worth noting that 29 out of 55 predictor genes were associated with expression in the brain according to enrichment analysis using DAVID on UniProt tissue expression categories (UP_TISSUE, EASE score P = 0.071, FDR 53.88). Also, hierarchical clustering of samples in P1 by the ASD55 predictor genes showed a clear distinction between ASD patients and controls (Fig. 3).

### Effect of other clinical and demographic factors on blood gene expression

In order to ensure that our predictor was robust for ASD classification, we reviewed the expression data for potential confounders. Among the demographic and clinical features, age at time of blood draw significantly influenced gene expression. Within the ASD group, age at blood collection was correlated within 382 genes at a significance level of *P*<0.001 (Spearman's rank correlation test, N = 66, corresponding FDR 0.018). Six KEGG pathways were significantly enriched with the 382 age-correlated genes in the P1 ASD population (Table S7). The carbon pool by folate pathway (KEGG ID: hsa00670) was the most significantly enriched with age-correlated genes (EASE score *P* = 4.6×10^−7^, FDR 5.2×10^−4^). The age-correlated genes in this pathway were *MTHFD1, TYMS, SHMT2, ATIC, DHFR, MTHFD1L*, and *GART*. The ASD55 genes were not significantly correlated with age except for *CNTRL* and *UTY*, which were correlated with age in patients but not controls. *UTY* was one of the 23 genes that were differentially expressed in both datasets (P1 and P2). In the P1 control group (N = 33), 163 genes correlated significantly with age, but none of the ASD55 genes were among them.

Several other clinical and developmental characteristics were also correlated with gene expression changes as summarized in Table 6. A positive personal history of developmental delay including a delay in hitting milestones such as sitting, crawling, walking, and speaking was associated with 12 genes including the aristaless related homeobox gene *(ARX). ARX* is a homeodomain transcription factor that plays crucial roles in cerebral development and patterning [45], and is implicated in X-linked intellectual disability [46]. *ARX* was not differentially expressed in the ASD group of P1 (*P* = 0.74); however, it was significantly down-regulated in the individuals with positive history of developmental delay (*P* = 0.00037, FDR 0.30).

**Table 6:** Genes significantly correlated with clinical features.

Medical and developmental history	Number of significant genes (p<0.001)	Significant genes
**Developmental delay**	12	*ARX,BMS1P1,C20orf196,CCDC18,IBTK,PNRC1,RHBDL2,TIGD1,TRIM4,ZNF37A,ZNF415,ZNF536*
**Learning disorders**	68	*ADRA1B,AKNAD1,ANKRD18A,ANKRD30A,APP,BOD1L,C20orf166-A, C6orf195,CA2,CACNG5,CAV2,CEP19,CHRM2,CLDN5,CNTNAP3,CRYGN,CXCL5,DDX11L2,ENSG00000217702,EPHA10,F13A1,FAM184B,FMO3,GFOD1,GGTA1P,GIF,GNG11,GSC2,HBEGF,HGD,HRCT1,IGSF11,IGSF22,ITPRIPL2,IZUMO1,KCNA1,KRT81,LCE1B,LOC126536,LYZL4,MECOM,MSH4,NME5,NPY,NR1H4,P2RX3,PACS2,PF4V1,PPFIA2,PPP3R1,RAX2,RNF17,RPL21P68,SCGN,SCN9A,SHH,SLC16A9,SLCO2B1,SMCR8,SYN2,TCTN2,TEAD1,TMIE,TRH,TXNRD2,VGLL3,WRB,ZNF652*
**Neurological disorders**	7	*FAM13A,LRRD1,PITX3,SH3PXD2B,SPRR4,SPZ1,TACR2,*
**Psychiatric disorders**	5	*CSTT,GPR111,HIP1,MED25,STX19*
**Gastrointestinal disorders**	5	*COL7A1,MARK1,PLA2G4C,SETMAR,TTR*
**Seizure disorders**	4	*GPR153,GSC2,MGC39545,PITX3*

In the P1 cohort, 9 patients with ASD were diagnosed with leaning disorders. Sixty-four genes were differentially expressed with regard to learning disorders (Positive History N = 9, Negative History N = 90, *P*<0.001, corresponding FDR 0.14). The calcium signaling pathway (KEGG ID: hsa04020) was significant (hypergeometric *P* = 0.023, FDR 0.19) due to *ADRA1B, CHRM2, PPP3R1*, and *P2RX3*. Another gene differentially expressed in patients with learning disorders, Synapsin 2 (*SYN2*), is a synaptic vesicle-associated protein that has been implicated in modulation of neurotransmitter release and in synaptogenesis. A brain gene expression study showed that *SYN2* was down-regulated in the prefrontal cortex of schizophrenic patients [47]. The differentially expressed genes that were correlated with other clinical conditions including psychiatric, neurological, gastrointestinal disorders, and seizure disorder are summarized in Table 6.

## Discussion

Prior studies have shown differentially expressed genes and miRNAs in brain [48]–[52] and blood [15]–[23] samples from patients with ASD. This study further examines gene expression and demonstrates the capability of blood gene expression profiling to distinguish ASD patients from controls, with an average accuracy of 72.5% in one population (the P1 cohort) and 72.7% in an independently collected validation population (the P2 cohort).

The classification performance in this study is encouraging, particularly as the two groups were heterogeneous and profiled using two different array-types. The classification of 73% of cases by expression profiling contrasts with the small percentage of ASD cases characterized by genetic mutations or structural variations to date. It also compares favorably to the performance of CMA, which, while high confidence, accounts for only 7–10% of cases of ASD. Together, these results suggest that gene expression signatures, which comprise multiple perturbed pathways, may serve as signals of genetic change suggestive of ASD in most patients. In this regard, this work parallels studies in neuropsychiatry where investigators have demonstrated that blood expression signatures are significantly different in schizophrenia [53], Alzheimer's disease [54], and bipolar disorder [55].

Although the transcriptomic connection between blood and brain is not well understood, numerous lines of evidence suggest that measurements in tissues that are not primarily involved in the disease process may reveal disease signatures. Several investigators have demonstrated differential expression of genes in peripheral white blood cells in disorders of the central nervous system [53]–[56]. To this point, Sullivan *et al.* [57] have established a shared expression profile between different CNS tissues and the blood suggesting the use of peripheral blood expression as a surrogate for the brain. Moreover, individual gene expression variations of multiple brain regions correlate well with those of blood in non-human primates [58]. Recently, gene expression profiles of lymphoblastoid cell lines were shown to distinguish between different forms of ASD caused by defined genetic lesions (Fragile X syndrome and chromosome 15q duplication) and normal controls [22], and small studies of patients phenotypically defined with ASD have shown differential expression of genes in their peripheral blood cells [20] and in the function of T cell subsets [19]. These results are mirrored by proteomic studies of serum, which suggest systematic differences between patients with ASD and controls [59]. As such, this evidence suggests that peripheral blood cells might be used as a surrogate for gene expression in the developing nervous system. Moreover, Glatt et al. recently reported results from an on-going longitudinal study of blood gene expression biomarkers in ASD and typically developing children [60]. They compared peripheral blood mononuclear cell gene expression profiles from ASD (37 AD and 23 PDD) with 68 non-cases – 27 samples from typically developing children and 41 samples from children who were initially evaluated as a potential risk group, but later found to be non-cases. Among the 134 differentially expressed genes found by Glatt et al., 5 genes—*ABHD3, COL4A3BP, MAPK14, PARP8*, and *ZNF763*—were also differentially expressed in our P1 dataset, and *ZNF763* was significant in the P2 data as well. The overlapping genes were all up-regulated in our datasets while the same genes were all down-regulated in Glatt et al. except for *ZNF763*, which was up-regulated in our two datasets P1 and P2, and in Glatt et al. It is possible that the effect of age on blood gene expression contributed to the gene expression changes being opposite for the common genes. A longitudinal follow-up study of the cohort of Glatt et al. would give us more conclusive results regarding the validity of blood gene expression markers at different age groups.

The biological pathways implicated by the differentially expressed genes identified in this study are of interest because some of the gene sets link to synaptic activity-dependent processes (i.e., long-term potentiation and neurotrophin signaling in Table 4), for which several ASD mutations have been found [40], [41]. Immune/inflammatory pathways were also identified in this analysis (e.g. chemokine signaling and Fc gamma R-mediated phagocytosis), which have been implicated in several studies of children with ASD compared to controls through CNS cytopathology [61], serum and CSF proteomics [59], as well as in cadaveric expression studies of the CNS [51].

According to OMIM, which covers most reported associations between diseases and genes [62], 6 of the ASD55 genes (11%) are known disease related genes. Among these 6 genes, *CREBBP* and *RPS6KA3* were associated with intellectual disability. Heterozygous mutation of *CREBBP* causes Rubinstein-Taybi syndrome [63], of which the core symptom is intellectual disability (MIM ID# 180849). Coffin-Lowry syndrome (MIM ID# 303600) is caused by mutations in *RPS6KA3* on chromosome Xp22.12, and is characterized by skeletal malformation, growth retardation, cognitive impairments, hearing deficit, and paroxysmal movement disorders [64].

There remain several potentially important limitations of this study. The two data sets were obtained at different times and the methods for RNA acquisition and microarrays used in P1 differed in part from those in P2. Also, the control population in P2 versus P1 differed in the clinics from which they were drawn, and the racial and ethnic backgrounds of the patient and control populations were not completely matched. This heterogeneity adds noise to the case vs. control comparison and conversely if the analysis utilized more homogeneous data sets, we would have expected improved accuracy. Despite these differences, the independent set reassuringly demonstrates the accuracy of the classifier. However, if ASD expression endophenotypes exist, we did not achieve sufficient sample size to discover them. The inability to identify subtypes within an autism cohort is not unusual, as it has also been seen in recent genotyping and copy number variation studies [27], [65]. Also, the data were collected after diagnosis and not as part of a longitudinal study of individuals. The application of these predictors to a prospective cohort would allow us to further assess their validity as a diagnostic and prognostic tool. Finally, our groups with ASD were compared to developmentally normal controls and not to individuals with other neurodevelopmental disorders. Nevertheless, the accuracy we have obtained in this study is a necessary first step towards a trial validating a set of predictive biomarkers.

In conclusion, this study of children with ASD describes a gene expression signature that shows promising accuracy in classifying children with ASD from controls. The ability of the ASD55 predictor to correctly classify ASD samples compares favorably to the DNA-based tests currently proposed for ASD diagnosis. The results presented here raise further questions that bear investigation but are outside this study's scope: At what age does this ASD55 signature manifest? Is it present at birth? Finally, we expect that larger studies can be used to determine whether particular characteristics of ASD can be classified or predicted from a gene expression signature (e.g. seizures and language delay) and thereby improve individualized treatment in the near future.

## Materials and Methods

### Blood gene expression profiling

Gene expression profiles of P1 were prepared using Affymetrix HG-U133 Plus 2.0 (U133p2) and those of P2 were profiled using Affymetrix Gene 1.0 ST (GeneST) arrays (Affymetrix, CA). Within the P1 data set, RNAs from 39 ASD and 12 control samples were isolated directly from whole blood using the RiboPure Blood Kit (Ambion). For all other blood samples, total RNA was extracted from 2.5 ml of whole venous blood using the PAXgene Blood RNA System (PreAnalytix) according to the manufacturer's instructions. Quality and quantity of these RNAs was assessed using the Nanodrop spectrophotometer (Thermo Scientific) and Bioanalyzer System (Agilent). Fragmented cRNA was hybridized to the appropriate Affymetrix array and scanned on an Affymetrix GeneChip scanner 3000. cRNA from both affected and normal control population groups was prepared in batches consisting of a randomized assortment of the two comparison groups.

### Processing of microarray data

Gene expression levels were calculated using Affymetrix Power Tools version 1.10 (Affymetrix, CA). We used the Probe Log Iterative ERror (PLIER) algorithm that includes a probe-level quantile normalization method for each microarray platform separately [66]. To match the probeset identifiers from the two different platforms used in this study, we used the Best Match subset (http://www.affymetrix.com/Auth/support/downloads/comparisons/U133PlusVsHuGene_BestMatch.zip) between the two as described in the Affymetrix technical note [67]. 29,129 out of 54,613 total probesets on U133p2 were best matched to 17,984 unique probesets of the GeneST array, and these matched probesets were used for the cross-platform prediction analysis. For the genes represented by more than two U133p2 probesets, we included the genes for which all probesets changed to the same direction.

To identify hidden confounders such as batch effect, we performed surrogate variable analysis (SVA) with null model for batch effect [68]. For the P1 dataset, SVA found 6 surrogate variables in residuals after fitting with the primary variable of interest, i.e., clinical diagnosis. The first surrogate variable significantly correlated with the year when the microarray profiling was performed. In the P2 dataset, a batch with 12 samples was grouped separately from the other 172 samples from a principal component analysis although none of the surrogate variables was correlated with the 12 outlier samples. We used the ComBat algorithm [69] to reduce the batch effects in P1 and P2 independently as the two array platforms are different in the design of probe sequences such that U133p2 array uses both perfect match (PM) and mismatch (MM) probes while GeneST array only has PM probes. All statistical analyses were performed with the ComBat corrected expression data.

### Statistical analysis for differentially expressed genes and enriched pathways

To identify differentially expressed genes in cases compared to controls, we used Welch's t-test for two group comparison, and one-way analysis of variance with Dunnett's *post hoc* tests to find significantly changed genes in AUT, PDDNOS, or ASP compared to the control group. To identify differentially expressed genes in the P2 dataset, the significance of diagnosis and gender was determined by two-way analysis of variance and follow-up Welch's t-test for each gender and Dunnett's *post hoc* tests for subtypes. We set the threshold for differential expression at nominal p-value<0.001. A general linear model was used to evaluate the significance of diagnosis, gender, age, and the other covariates. We corrected p-values for multiple comparisons by calculating a false discovery rate (FDR) [70]. We used Fisher's exact test for categorical data. Spearman's rank correlation coefficients were calculated to evaluate correlation between continuous phenotypic variables such as age at blood drawing and the expression level of each gene. The significance of correlation was determined using Fisher's *r*-to-*z* transformation. Enriched biological pathways with predictor genes were found using the DAVID functional annotation system [71]. For significant KEGG pathways, we calculated the robust Mahalanobis distance of each individual from the common centroid of all cases and controls to find outliers using the minimum covariance determinant estimator [72]. A quantile of the chi-squared distribution (e.g., the 97.5% quantile) was used as a cut-off to define outliers, because for multivariate normally distributed data the Mahalanobis distance values are approximately chi-squared distributed. These outliers can be interpreted as biologically distinct subgroups for each pathway. All statistical analysis was performed using the R statistical programming language [73], and robust multivariate outlier analysis was performed using the chemometrics R library package [72].

### Statistical prediction analysis

We performed prediction analysis in the following sequential steps; 1) ranking genes for predictor selection, 2) setting up a cross-validation strategy in the training set, 3) tuning parameters and building prediction models, and 4) predicting a test set, and evaluating prediction performances (Fig. S4). First, all genes were ranked by AUC. We selected the top 10 genes from the ranked list to build a prediction model with a partial least square (PLS) method in the P1 dataset using a repeated leave-group out cross-validation (LGOCV) strategy, then repeated the same procedure with the top N genes incremented by 5 up to 390. For each prediction model using the top N genes, all P1 samples (N = 99) were divided to 80% (a train set) and 20% (a test set), keeping the proportion of ASD cases and controls the same in each set. This step was repeated 100 times to estimate robust prediction performance (i.e., outer cross validation). To optimize each prediction model further, an inner cross-validation approach was deployed where 80% of the samples served as an inner train set, and 20% were used as an inner test set. The inner cross-validation procedure was repeated 100 times to find optimal tuning parameters for the specific prediction algorithm used. For each prediction model with the top N genes, a total of 10,000 predictions (i.e., 100 repeated LGOCVs ×100 inner cross-validations) were made.

For each sample in a test set, the model predicts the probability of being classified as ASD. Thus, the number of false positives among positive predictions changes with the threshold. Overall prediction accuracy was calculated as (the number of true positives+the number of true negatives)/N, where N was the total number of samples in a dataset. Sensitivity, specificity, positive predictive value, and negative predictive value were presented as standard measures of prediction performance with AUC. The ROC curve summarizes the result at different thresholds.

To find the best performing prediction model with the minimum description length, we compared AUCs between prediction models using the top N genes. The mean AUCs improved gradually with increasing model complexities. However, we could identify the most stable prediction model by calculating the coefficient of variation of AUCs with 100 trials of outer cross validations. We tested 5 additional prediction methods; Logistic regression, Naïve Bayes, k-Nearest Neighbors, Random Forest, and Support Vector Machine using 55 genes with 5 fold LGOCV strategy (Table S5). Statistical prediction analysis was performed using the caret [74] and RWeka [75] R library packages.

### Quantitative RT-PCR validation

A total of 12 genes using 30 ASD and 30 control samples from the P1 population were run in replicates of four on the Biomark real time PCR system (Fluidigm, CA) using nanoliter reactions and the Taqman system (Applied Biosystems, CA). We were limited to 60 samples because the other 39 samples did not have enough RNA for qRT-PCR. Following the Biomark protocol, quantitative RT-PCR (qRT-PCR) amplifications were carried out in a 9 nanoliter reaction volume containing 2× Universal Master Mix (Taqman), taqman gene expression assays, and preamplified cDNA. Pre-amplification reactions were done in a PTC-200 thermal cycler from MJ Research, per Biomark protocol. Reactions and analysis were performed using a Biomark system. The cycling program consisted of an initial cycle of 50°C for 2 minutes and a 10 min incubation at 95°C followed by 40 cycles of 95°C for 15 seconds, 70°C for 5 seconds, and 60°C for 1 minute. Data was normalized to the housekeeping gene *GAPDH*, and expressed relative to control. All primers used for the 12 genes are listed in Table 2.

## Supporting Information

Figure S1 **Principal component analysis of 285 blood gene expression profiles.** Global gene expression profile of the Training set (P1) and the Validation set (P2) samples. After selecting the best-matching probe sets between two Affymetrix microarray platforms (see Methods), principal component analysis was performed. We applied the ComBat method to reduce batch effect for each dataset. All samples from P1 and P2 were projected to two-dimensional space of the first (PC1) and the second (PC2) principal components after centering and scaling expression levels in each dataset. 36.5% of overall variance was explained by PC1 and PC2. We did not find global gene expression difference between ASD cases and controls.
(TIF)

Figure S2 **Selecting the predictor genes using repeated cross validations.** Our prediction model selection procedure consisted of three nested loops as illustrated in **Fig. S3**. The outer-most loop was the selection of the top N genes (from 10 to 395 incremented by 5) from the AUC ranked gene list. The second loop was a leave-group out cross validation approach, where 80% of samples were randomly selected as a train set, while maintaining the proportion of each diagnostic class. This step was repeated 100 times for each list of the top N genes. The inner-most loop was used to optimize the parameters that were specific to machine learning methods used for a train set from an outer loop. This parameter tunings were repeated 100 times by randomly selecting 80% of the train set samples. The prediction performance was estimated using AUC. We found the mean AUCs improved gradually when we increased the number of genes to build more complex prediction models (left); however, the top 55 genes prediction model performed significantly better than the 50 gene model (t-test *P* = 0.00031) and also presented the smallest coefficient of variation from 100 repeated cross validations (right).
(TIF)

Figure S3 **Predictor gene selection and model building procedure.**
(TIF)

Figure S4 **Overlap between differentially expressed genes for each diagnostic subgroup (ASP, PDD, AUT) in P1.** Only one gene, PTPRE, was found in common as significant genes for each diagnostic subgroup vs. control. And 36 genes were common between AUT vs. control (177 significant genes) and PDDNOS vs. control (56 significant genes).
(TIF)

Table S1 **Differentially expressed genes in P1.** We used Welch's t-test for two groups comparison, and one-way analysis of variance with Dunnett's *post hoc* tests to find significantly changed genes in autistic disorder (AUT), PDD-NOS (PDDNOS), or Asperger's disorder (ASP) compared to control group. We corrected p values for the multiple comparisons by calculating a false discovery rate (FDR).
(XLS)

Table S2 **Differentially expressed genes in P2.** We used Welch's t-test for the comparison between ASD cases and controls. To identify differentially expressed genes in P2 dataset, significance of diagnosis (p(Dx)) and gender (p(Gender)) was determined by two-way analysis of variance (ANOVA) and follow-up Welch's t-test for each gender. p(Dx*Gender) denotes the interaction between diagnosis and gender effects for significance. A total of 469 unique genes were differentially expressed (*P*<0.001, corresponding FDR 0.023) as there were transcripts without official gene symbols (i.e., – in *Gene* field) and several genes have multiple Affymetrix IDs.
(XLS)

Table S3 **Top 6 clusters of Gene Ontology biological process terms enriched for differentially expressed genes in P1 data set.**
(XLS)

Table S4 **The predictor genes for final prediction model.** The differentially expressed genes were ranked by AUC, and top 55 genes were selected to build the final prediction model. Affymetrix IDs represent the transcript IDs of Gene ST 1.0 array. Welch's t-tests were used to calculate p-values, and false discovery rates (FDR) were calculated as described in Storey and Tibshirani.
(XLS)

Table S5 **Prediction performance of ASD55 using various machine learning algorithms.** ASD55 denotes the genes in a classifier developed on P1 with 55 genes (**Table S4**). The average prediction performances from 100-repeated leave-group out cross validations using the P1 dataset are shown. For each prediction instance, 20% of ASD cases (N = 13) and 20% of controls (N = 7) were randomly selected for a testing set, and the other 80% of samples served as a training set. This procedure was repeated 100 times to calculate the average performance of ASD55 with 6 machine learning algorithms listed below. The overall performance of PLS was comparable to the other 5 methods. The sensitivities were relatively higher than the specificities across different methods except for the Naïve Bayes classifier. (AUC: Area under the receiver operation characteristics curve, ACC: Accuracy, SENS: Sensitivity, SPEC: Specificity, PPV: Positive Predictive Value, NPV: Negative Predictive Value).
(XLS)

Table S6 **Functional enrichment of genes in ASD55.** The term categories are presented as defined in DAVID.
(XLS)

Table S7 **Pathways enriched with age-correlated genes in ASD.**
(XLS)
