# Supplementary figures and images for: Characteristics and Predictive Value of Blood Transcriptome Signature in Males with Autism Spectrum Disorders
Source: PLoS One. 2012 Dec 5;7(12):e49475. doi: 10.1371/journal.pone.0049475 (PMC3515554; doi:10.1371/journal.pone.0049475)

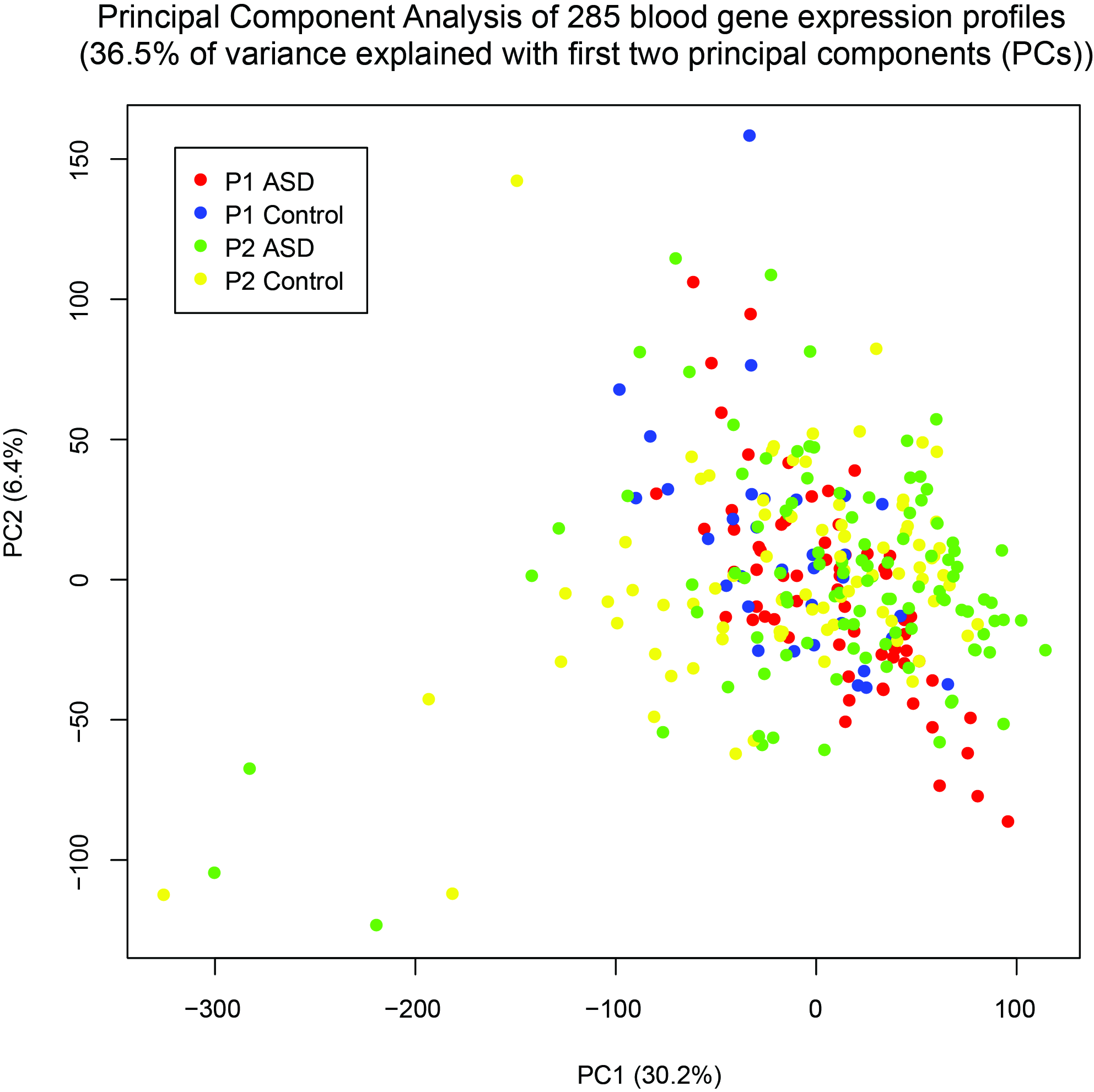

Supplement: Figure S1 — Principal component analysis of 285 blood gene expression profiles. Global gene expression profile of the Training set (P1) and the Validation set (P2) samples. After selecting the best-matching probe sets between two Affymetrix microarray platforms (see Methods), principal component analysis was performed. We applied the ComBat method to reduce batch effect for each dataset. All samples from P1 and P2 were projected to two-dimensional space of the first (PC1) and the second (PC2) principal components after centering and scaling expression levels in each dataset. 36.5% of overall variance was explained by PC1 and PC2. We did not find global gene expression difference between ASD cases and controls. (TIF) [file pone.0049475.s001.tif]

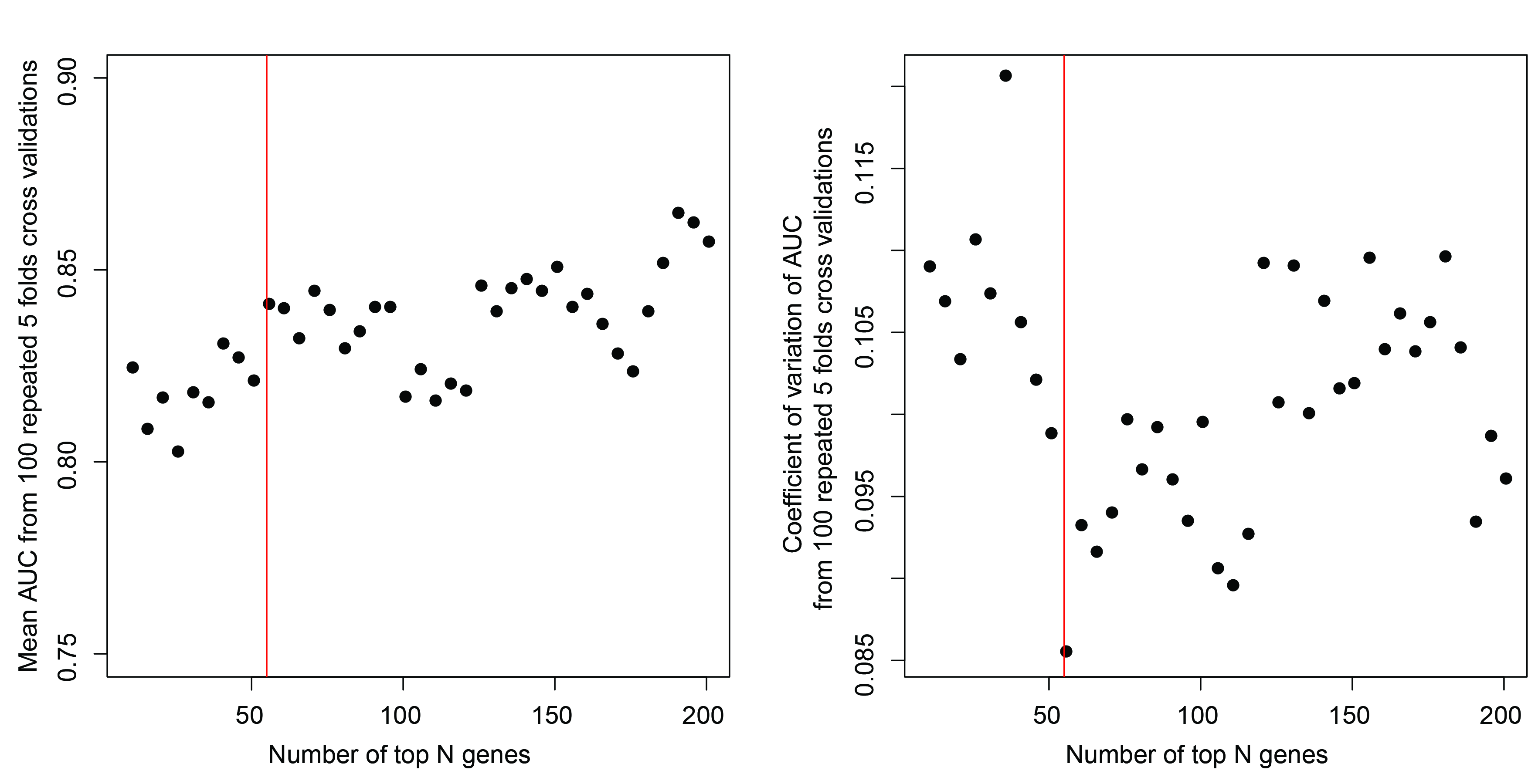

Supplement: Figure S2 — Selecting the predictor genes using repeated cross validations. Our prediction model selection procedure consisted of three nested loops as illustrated in Fig. S3. The outer-most loop was the selection of the top N genes (from 10 to 395 incremented by 5) from the AUC ranked gene list. The second loop was a leave-group out cross validation approach, where 80% of samples were randomly selected as a train set, while maintaining the proportion of each diagnostic class. This step was repeated 100 times for each list of the top N genes. The inner-most loop was used to optimize the parameters that were specific to machine learning methods used for a train set from an outer loop. This parameter tunings were repeated 100 times by randomly selecting 80% of the train set samples. The prediction performance was estimated using AUC. We found the mean AUCs improved gradually when we increased the number of genes to build more complex prediction models (left); however, the top 55 genes prediction model performed significantly better than the 50 gene model (t-test P = 0.00031) and also presented the smallest coefficient of variation from 100 repeated cross validations (right). (TIF) [file pone.0049475.s002.tif]

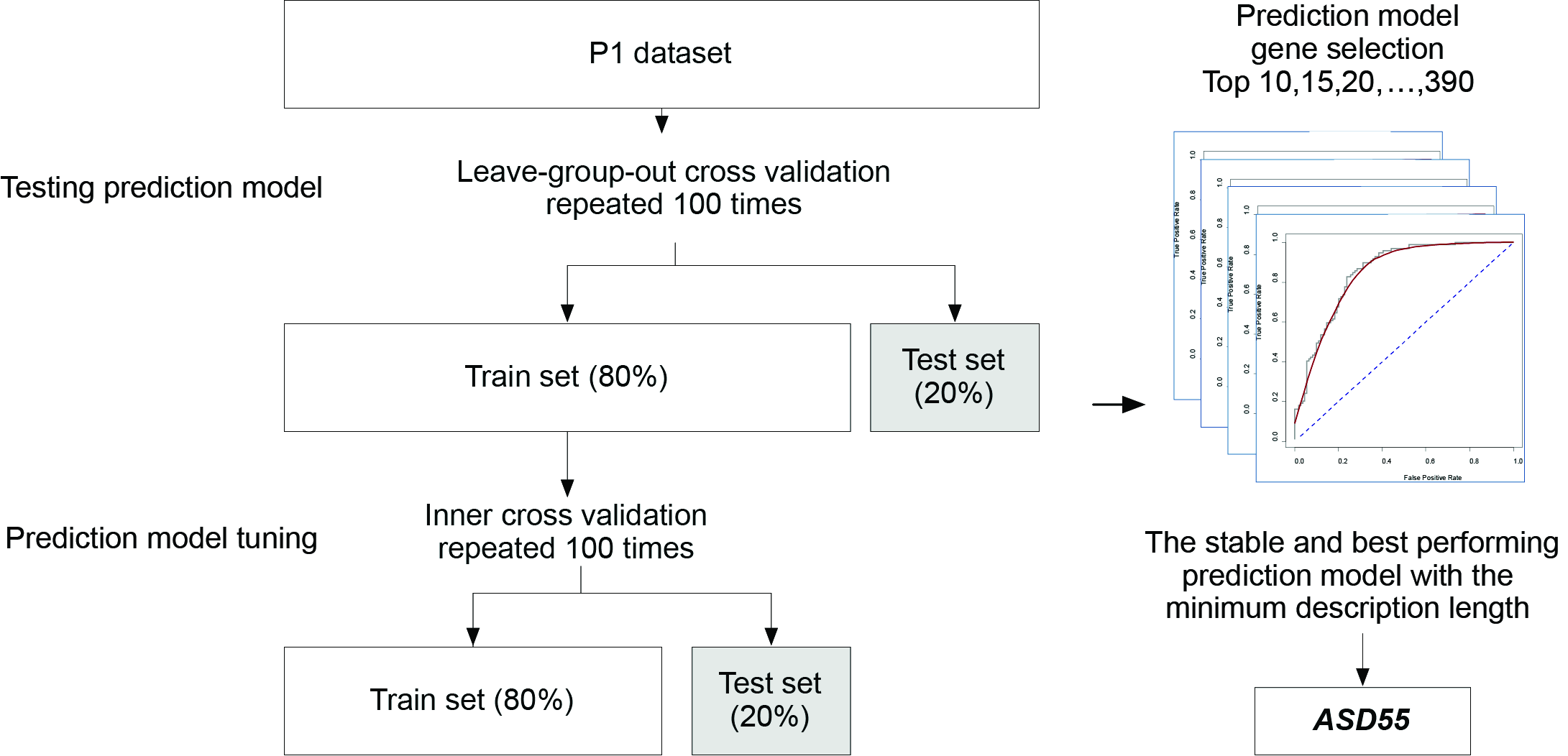

Supplement: Figure S3 — Predictor gene selection and model building procedure. (TIF) [file pone.0049475.s003.tif]

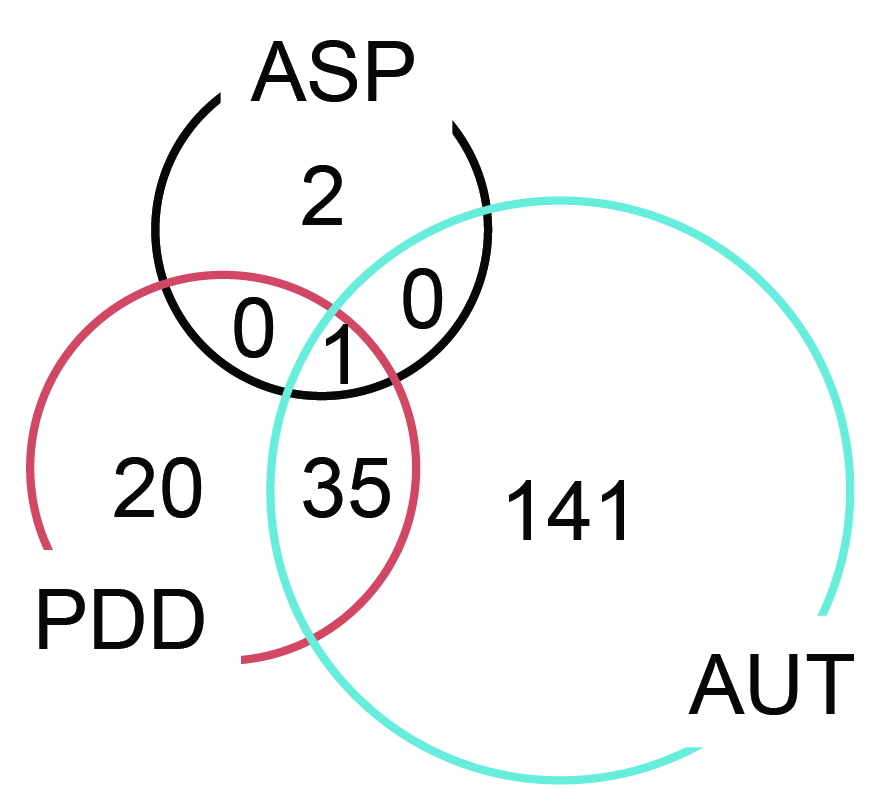

Supplement: Figure S4 — Overlap between differentially expressed genes for each diagnostic subgroup (ASP, PDD, AUT) in P1. Only one gene, PTPRE, was found in common as significant genes for each diagnostic subgroup vs. control. And 36 genes were common between AUT vs. control (177 significant genes) and PDDNOS vs. control (56 significant genes). (TIF) [file pone.0049475.s004.tif]
